# Understanding the Multifaceted Role of Neutrophils in Cancer and Autoimmune Diseases

**DOI:** 10.3389/fimmu.2018.02456

**Published:** 2018-11-09

**Authors:** Xu Wang, Lin Qiu, Ziyi Li, Xiang-Yang Wang, Huanfa Yi

**Affiliations:** ^1^Central laboratory of Eastern Division, The First Hospital of Jilin University, Changchun, China; ^2^Institute of Immunology, Jilin University, Changchun, China; ^3^National-local Joint Engineering Laboratory of Animal Models for Human Diseases, Changchun, China; ^4^Department of Neurology, The First Hospital of Jilin University, Changchun, China; ^5^Department of Human and Molecular Genetics, Virginia Commonwealth University, Richmond, VA, United States; ^6^Massey Cancer Center, Virginia Commonwealth University, Richmond, VA, United States

**Keywords:** neutrophils, cancer, N1/N2, autoimmune diseases, immune tolerance

## Abstract

Neutrophils are one of the first immune cell types that are recruited to injury and infection site. As a vital component of the immune system, neutrophils are heterogeneous immune cells known to have phagocytic property and function in inflammation. Recent studies revealed that neutrophils play dual roles in tumor initiation, development, and progression. The multifunctional roles of neutrophils in diseases are mainly due to their production of different effector molecules under different conditions. N1 and N2 neutrophils or high density neutrophils (HDNs) and low density neutrophils (LDNs) have been used to distinguish neutrophils subpopulations with pro- vs. anti-tumor activity, respectively. Indeed, N1 and N2 neutrophils also represent immunostimulating and immunosuppressive subsets, respectively, in cancer. The emerging studies support their multifaceted roles in autoimmune diseases. Although such subsets are rarely identified in autoimmune diseases, some unique subsets of neutrophils, including low density granulocytes (LDGs) and CD177^+^ neutrophils, have been reported. Given the heterogeneity and functional plasticity of neutrophils, it is necessary to understand the phenotypical and functional features of neutrophils in disease status. In this article, we review the multifaceted activates of neutrophils in cancer and autoimmune diseases, which may support new classification of neutrophils to help understand their important functions in immune homeostasis and pathologies.

## Summary points

- Neutrophils play multifaceted functions under different pathological conditions by releasing various effector molecules and cytokines.
- Neutrophils are more heterogeneous than previously thought and different subpopulations have distinct activities in diseases.
-Neutrophils in cancer could be divided into N1 and N2 subsets, or high density neutrophils, and low density neutrophils based on functional characteristics.
- Neutrophils in certain autoimmune diseases may also be classified into different subsets, with low density granulocytes (LDGs) representing pro-inflammatory neutrophils and Gr-1^high^ or CD177^+^ neutrophils exhibiting anti-inflammatory effects.

## Introduction

Neutrophils are the most abundant leukocytes in the circulating system, making up 50–70% of the whole white blood cells in human (1, 2). They constitute the first line of defense and protect the host from pathogen assaults via multiple mechanisms including phagocytosis, release of granules, production of cytokines, and formation of neutrophil extracellular traps (NETs) (3). Neutrophils are not only an important component of innate immunity, but also participate in regulation of adaptive immunity through interplays with various adaptive immune cells. In addition to the host defense, neutrophils are also involved in the pathogenesis of many diseases, including cancer and autoimmune disorders (2).

Although neutrophils have long been found to be present in different type of tumors, these tumor-associated neutrophils (TANs) were believed to be functionally neutral due to their short lifespan (2). Increasing studies over the past few years began to reveal the differential roles of neutrophils in cancer. Only recently, the level of neutrophils in the tumor tissues was considered as a marker for a poor prognosis of cancer patients (4). According to their functions, TANs are divided into two subgroups with anti-tumor (N1) or pro-tumor (N2) activity (5–7), which is similar to tumor-associated macrophages (8, 9). Recently, it was suggested that circulating neutrophils can also be classified into high density neutrophils (HDNs) and low density neutrophils (LDNs), which functionally mirrors N1 and N2 neutrophils, respectively (10, 11).

While neutrophils have also been reported to show multifaceted functions in many autoimmune diseases (12–17), few researchers have attempted to distinguish subpopulations of neutrophils in autoimmune diseases. Most immune cells, including T cells, dendritic cells, and macrophages have been classified into different subsets that exert different or even opposite roles in different disease contexts (18–21). In this article, we summarize our current understanding of neutrophils, the most abundant leukocytes in circulation, and their multifaceted functions in different diseases, particularly autoimmune conditions. We describe the various effector molecules produced by neutrophils that define its functions in a disease-specific context, which may provide some insights into the potential classification of neutrophils.

## The diversity of neutrophils in cancers

Cancer cells can produce various chemokines and cytokines, which recruit neutrophils to the tumor milieu (22, 23). Although neutrophils have previously thought to be terminally differentiated cells due to their short life span (24, 25), the plasticity of neutrophils has been unveiled in the recent years. These cells may be divided into different subsets based on their differential effects on cancer initiation, development, and progression. Neutrophils in cancer, also known as tumor associated neutrophils (TANs), are functionally classified as tumor-suppressing N1 or tumor-promoting N2 phenotype. Alternatively, they can be divided into high density neutrophils (HDNs) and low density neutrophils (LDNs) based on the density of circulating neutrophils in cancer patients.

N1 and N2 subpopulations of TANs display distinct functions in cancer. N1 neutrophils have potent anti-tumor activity mainly due to their release of pro-inflammatory or immunostimulatory cytokines, such as interleukin (IL)-12, tumor necrosis factor (TNF)-α, CCL3, CXCL9, CXCL10, which facilitates recruitment and activation of CD8^+^ T cells (26, 27). In contrast, N2 neutrophils have strong immunosuppressive and tumor-promoting activity, including promotion of tumor angiogenesis, invasion and metastases via various factors, such as hepatocyte growth factor (HGF) (28), oncostatin M (6), reactive oxygen species (ROS) (29), reactive nitrogen species (RNS) (29), matrix metalloproteinase (MMPs) (30), and neutrophils elastase (NE) (4, 30). TANs were shown to locate at the margin of tumor site in early stage cancer, but they can massively infiltrate into the center of tumor at late stage (31). Research in mouse lung carcinoma and mesothelioma models suggests that TANs have a tumor-suppressing N1 phenotype at the early stage of tumor, whereas they convert into a tumor-promoting N2 phenotype during tumor progression (31). Such a phenotypic transformation may be induced by the factors produced by cancer cells and/or other immune cells in the tumor microenvironment. Recent studies have shown that the cytokine TGF-β and type I interferons are major factors involved in polarization of neutrophils. In the presence of TGF-β, neutrophils are skewed toward an N2 phenotype, whereas the blockade of TGF-β facilitates neutrophil development into an N1 phenotype (32). In contrast, type I interferons polarize neutrophils to an N1 phenotype while the impaired type I interferon signal results in polarization of neutrophils to an N2 phenotype (33). A recent study reported that angiotensin converting enzyme inhibitors (ACEI) or angiotensin II type I receptor (AGTR1) antagonist also promoted an N1 phenotype of neutrophils, which was association with inhibition of tumor growth. However, addition of angiotensin II reversed this process (34). Furthermore, ACEI treatment resulted in a reduction of serum TGF-β in tumor-bearing mice, suggesting that angiotensin II regulates neutrophils polarization through induction of TGF-β and further underscores an important role of TGF-β in N1-N2 polarization.

HDNs and LDNs represent another classification for circulating neutrophils in cancer paients. Density gradient centrifugation is the most common and validated approach to separate mononuclear and polymorphonuclear leukocytes (10). Mature neutrophils were thought to exist in the high density sedimentary fraction of leukocytes with segmented nucleus (35). However, recent findings showed that some neutrophils also exist in the low density fraction (11, 36). In addition to classification of HDNs and LDNs in blood, LDNs can further be divided into mature and immature populations based on the shape of their nucleus. The latter is also known as granulocytic-myeloid derived suppressor cells (G-MDSCs), which have a characteristic of banded or ring-shaped nucleus (37–39). Compared with HDNs, LDNs show a larger cellular size, reduced anti-tumor cytotoxicity, decreased phagocytic activity, impaired migratory capacity, and less oxidative burst, instead, these cells exhibit highly anti-inflammatory and tumor-promoting activity (11). In this context, HDNs are functionally similar to N1 neutrophils, while LDNs resemble N2 neutrophils. Interestingly, HDNs can be converted into LDNs by TGF-β (11), indicating the plasticity of these cells. However, this phenomenon was only observed in HDNs from tumor-bearing mice not those from tumor-free mice (11), suggesting that HDNs may need to be primed before their conversion into LDNs in the presence of TGF-β. Given the functional similarity of LDNs and N2 neutrophils, they are likely to belong to the same subpopulations of neutrophils that are present in different sites. However, it is difficult to test this possibility due to a lack of validated surface markers currently available to define N2 neutrophils or LDNs. Therefore, further investigation is necessary to understand the developmental relationship between N1/N2 neutrophils and HDNs/LDNs and, more importantly, to phenotypically identify these different subtypes of neutrophils as well as signals or factors involved in regulation of their polarization.

## The dual roles of neutrophils in cancers

**Figure 1:**
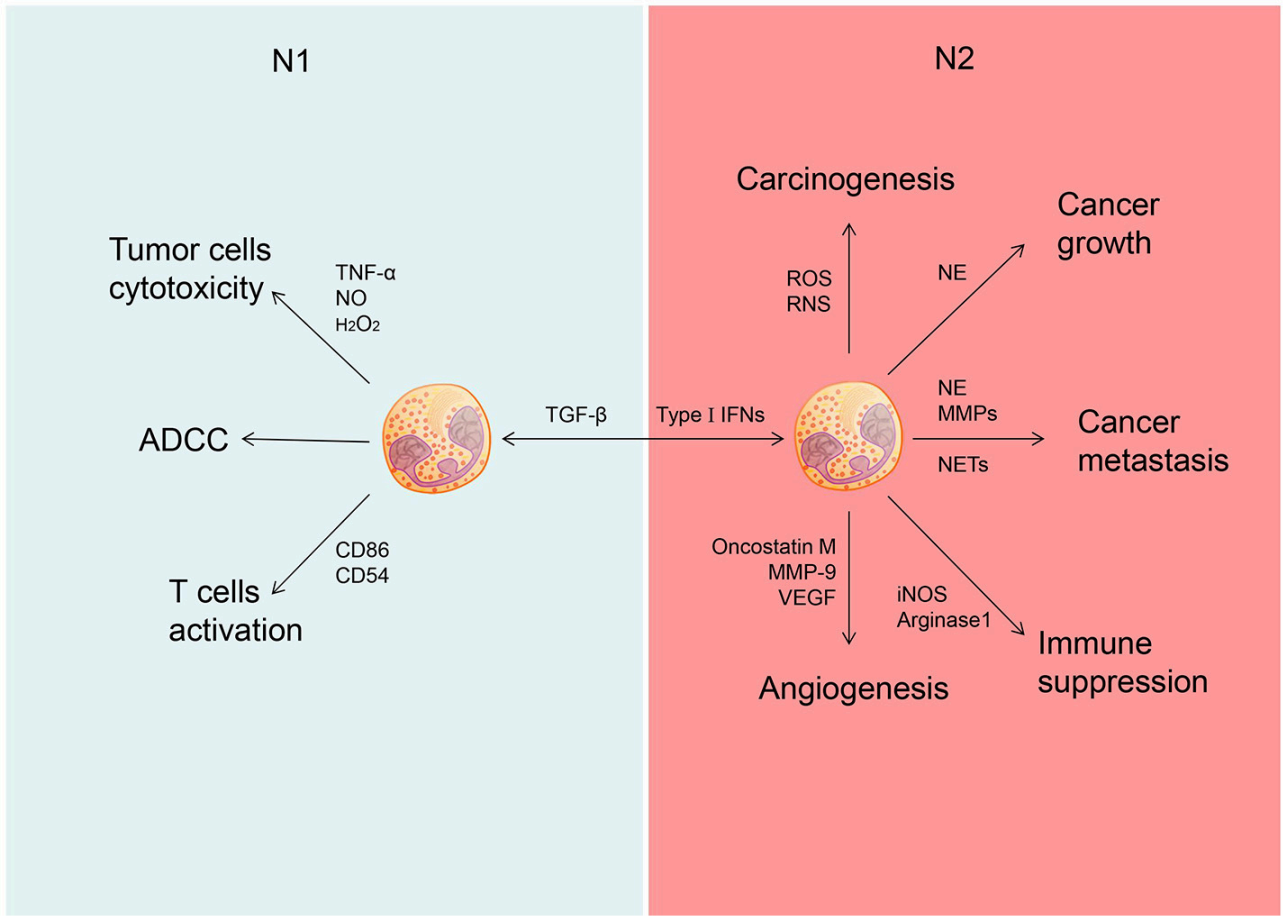
Different role of N1 and N2 neutrophils in cancer. Neutrophils could be polarized into N1 phenotype under the induction of TGF-â and polarized into N2 phenotype under the induction of type I IFNs. N1 neutrophils could inhibit the development of cancer through tumor cells cytotoxicity, ADCC and activate T cells. N2 neutrophils could promote the development of cancer through promoting carcinogenesis, tumor growth, cancer metastasis, and cancer angiogenesis as well as suppress immunity.

As an important immune cell type, neutrophils are present in circulation and tumor milieu. These cells display either pro- or anti-tumor activity in a context-specific manner (Figure 1).

The anti-tumor activity of neutrophils is executed mainly through antibody-dependent cellular cytotoxicity (ADCC) and target-specific antibody cytotoxicity (40, 41). Many potential cytotoxic mediators produced by neutrophils, including TNF-α, NO, and H_2_O_2_, can contribute directly to their tumor-suppressive activity (6, 31). Certainly cytokines, such as GM-CSF and IFN-γ, upregulate co-stimulatory molecules (e.g., CD86, CD54, OX40L, and 4-1BBL) on neutrophils at the early stage of tumorigenesis, which enable these cells to function as antigen-presentation cells for priming proliferation and activation of tumor-reactive T cells (42, 43).

The role of neutrophils have been described in various cancer types, including colorectal cancer (44, 45), glioblastoma (46), hepatocellular carcinoma (47), renal cell carcinoma (48), melanoma (49, 50), pancreatic ductal carcinoma (51), and head and neck cancer (52). Some studies have shown that increased number of neutrophils in tumor tissue or elevated ratio of neutrophil vs. lymphocyte in peripheral blood is associated with a poor prognosis of cancer patients (45–49, 51, 52). More specifically, neutrophils exhibit their tumor-promoting effect by facilitating tumor initiation, invasion, angiogenesis, and metastasis. Firstly, neutrophils can promote tumor initiation through production of reactive oxygen species (ROS) and reactive nitrogen species (RNS). These reactive radicals are likely to cause DNA damage and genetic instability in some models of chemical induced carcinogenesis (29). In addition, neutrophils accelerate tumor growth and progression by secreting a variety of mediators (4). Neutrophils produce certain growth factors, such as hepatocyte growth factor (HGF), to enhance invasion of human pulmonary adenocarcinoma cells (28). Interestingly, HGF induces release of NO by neutrophils in a autocrine fashion to kill cancer cells, suggesting a negative feedback loop involved in interplays between neutrophils and transformed cells during tumor progression (53). Neutrophils can also release oncostatin M and matrix metalloproteinase (MMP-9) to induce production of vascular endothelial growth factor, which can promote angiogenesis and invasion of tumor cells in multiple cancer models (54–56). Furthermore, NE and MMPs released by neutrophils promote tumor progression by remodeling tumor extracellular matrix (ECM) (30). Similar to G-MDSCs, neutrophils can also produce arginase 1 to inhibit effector function of T cells (57). In patients with gastric cancer, neutrophils display T cell suppressive activity in association with surface expression of the immune checkpoint molecule PD-L1or programmed death-ligand 1, which is activated by GM-CSF via the JAK and STAT3 signaling pathways (54–56).

Neutrophils extracellular traps (NETs) are special structure formed by neutrophils, and consist of chromatin and antimicrobial granule proteins, including NE, cathepsin G, and myeloperoxidase (MPO). The presence of NETs within tumors was reported to correlate with poor outcomes of cancer patients (52). NETs can execute their tumor-promoting activity by facilitating proliferation and inhibiting apoptosis of cancer cells (4). Although the precise mechanism remains unclear, this pro-tumor effect of NETs is believed to involve several tumor-promoting molecules, e.g., NE, cathepsin G and MMP-9 (58). NETs can also enhance adhesion of circulating tumor cells to promote metastasis of lung carcinoma (49). Of note, NETs was also shown to reduce the threshold of T cell activation through direct contact and/or TCR signaling, thereby promoting an adaptive immune response that may help eliminate cancer cells (59). However, the definite evidence supporting NETs-mediated anti-tumor activity is still lacking.

## Neutrophils in autoimmune diseases

Accumulating evidence supports the involvement of neutrophils in pathogenesis of many autoimmune diseases. They can influence autoimmune processes either directly via various effector molecules or indirectly through interactions with other immune cells (Table 1). Indeed, neutrophils are believed to be a major cause for induction of autoantibodies in certain autoimmune diseases (2). It is now recognized that neutrophils display phenotypic or functional abnormalities in diverse autoimmune diseases.

**Table 1:** Neutrophils in autoimmune diseases.

**Diseases**	**Species**	**Effector molecules**	**Functions**	**References**
MS	Mouse	ROS	Destructing blood-brain-barrier	(60, 61)
	Mouse	MPO	Destructing blood-brain-barrier	(62)
SLE	Human	IFN-á	Damaging vascular endothelia	(13)
	Human	NETs	Killing endothelial cells and activating plasmacytoid dendritic cells	(14, 63)
RA	Mouse	PADI 4	Related to the generation of autoantibodies	(15)
	Human	TNF	Attracting T cells	(64)
	Human	B-lymphocyte stimulator	Activating B cells	(65, 66)
	Mouse	ROS	Decreased ROS levels is associated with autoimmune and chronic inflammation	(67, 68)
	Human	MMPs	Destructing cartilage	(69, 70)
	Mouse	RANKL	Resorbing bone	(71)
T1D	Human	NE	Related to the generation of â-cells antigens autoantibodies	(72)
	Human	Proteinase 3	Related to the generation of â-cells antigens autoantibodies	(72)
	Mouse	ROS	Destructing pancreatic â-cells	(73)
	Mouse	CRAMP	Activating plasmacytoid dendritic cells	(16)
IBD	Mouse	ROS	Killing bacteria and damaging intestinal mucusa	(74, 75)
	Human	MMP-9	Degrading the extracellular matrix and vascular repairement	(76, 77, 78)
	Human	NE	Disrupting intestinal barrier	(79, 80)
	Mouse	IL-22	Repairing epithelial integrity and resoling colitis	(81)

### Multiple sclerosis

Study of experimental autoimmune encephalomyelitis (EAE), a widely used mouse model of human multiple sclerosis (MS) showed that the number of neutrophils increased significantly at the acute phase but declined at the remission phase in the lesion (82). Administration of anti-Ly6G or anti-Gr-1 antibody to deplete neutrophils limits EAE development, suggesting that neutrophils may play a detrimental role in EAE or MS. In contrast, use of G-CSF to promote recruitment and activation of neutrophils can exacerbate EAE (83). A recent study showed that the ratio of neutrophil vs. lymphocyte may be a predictor for the progression of disability in patients with MS (12). Indeed, neutrophils from the peripheral blood of MS patients exhibit an inflammatory phenotype with increased degranulation, ROS production and NETs formation (84, 85). These neutrophils also show reduced apoptosis, which may contribute to the chronic inflammation and repeated relapse of MS (85). In addition, they can facilitate the disease pathogenesis by compromising blood-brain-barrier (BBB) and inducing oxidative stress via generation of ROS (60, 61). BBB destruction by neutrophils is likely to result from increased production of myeloperoxidase (MPO) because inhibition of MPO restores integrity of BBB and ameliorates severity of disease (62).

Despite the potential harmful effect of neutrophils on MS, Gr-1^high^ neutrophils from central nervous system of EAE mice were shown to significantly suppress proliferation of myelin-reactive T cells, which is dependent on IFN-γ production (86), suggesting the existence of a negative feedback pathway that limits the function of auto-reactive T cells in EAE. It is possible that Gr-1^high^ neutrophils maybe a subset of neutrophils with disease-protective activity in EAE. Given that G-MDSCs were previously characterized as CD11b^+^Gr-1^high^ immature cells in mice (87), these T cell-suppressive Gr-1^+^ neutrophils observed in EAE mice may represent a subpopulation of G-MDSCs. In this context, it is reasonable to believe that the majority of neutrophils in EAE and MS are disease-promoting highly pro-inflammatory cells, and those with T cell-suppressive activity, e.g., Gr-1^+^ neutrophils or G-MDSCs, are unable to limit exacerbated inflammation to impact on disease progression. However, experimental evidence is required to support this possibility.

### Systemic lupus erythematosus

An increase of a subpopulation of neutrophils, known as low density granulocytes (LDGs), was reported in systemic lupus erythematosus (SLE) many years ago (88). These cells display enhanced pro-inflammatory activity and increased synthesis of type I interferons (89). Intriguingly, normal high density neutrophils, upon incubation with the plasma from SLE patients, can be converted into LDGs. This decrease in their density may be caused by activation of normal neutrophils by certain soluble factors (e.g., immune complexes and complements), which is known to be associated with neutrophils degranulation and increased cell size (88). LDGs may promote the development of SLE by enhancing apoptosis of endothelial cells and impairing vascular repair. In this context, LDGs are also associated with an increased risk of vascular injury in SLE due to their endothelial cytotoxicity. LDGs also participate in the pathogenesis of SLE through increased NETs formation (63, 90). It was reported that the blockade of NETs formation alleviates the severity of disease (91). In this case, NETs not only can kill directly endothelia cells (63), but also enhance the production of inflammatory cytokines, including IL-1β and IL-18,via activation of the nucleotide-binding oligomerization domain-like receptors protein 3 (NLRP3) inflammasome in macrophages and promotion of IFN-α synthesis by plasmacytoid dendritic cells (pDC) (14). In addition, LDGs from SLE patients produce higher levels of pro-inflammatory cytokines, such as IFN-α and TNF-α, compared to autologous HDNs. However, the phagocytic capability of LDGs is significantly reduced (13), which is similar to the LDNs in cancer. Compared with LDNs from renal cell carcinoma patients, LDGs in SLE patients contains more percentage of immature neutrophils (10 vs. 40%) (92). While LDNs in cancer express higher levels of CD11b and CD66b than HDNs, there is no difference in the levels of these activating markers between LDGs and HDGs in SLE (13, 92). Together, LDGs with a pro-inflammatory phenotype play a deleterious role in SLE development. While LDGs and LDNs in the circulations of cancer patients share similar phagocytic capability, LDNs are shown to have a distinct anti-inflammatory phenotype, suggesting that the density of neutrophils may not be a suitable marker to classify neutrophils subsets in different disease settings.

### Rheumatoid arthritis

It has been shown that neutrophil-to-lymphocyte ratio (NLR) correlates with the disease activity of rheumatoid arthritis (RA) (93–95). When combined with platelet-to-lymphocyte ratio, it can serve as a prognostic biomarker for RA (96). The detrimental role of neutrophils in RA may result from their association with the production of autoantibodies against citrullinated peptides, which is supported by finding that neutrophils in inflamed joints express peptidylarginine deiminase (PADI) 4 enzyme capable of catalyzing the citrullination of arginine (15). Neutrophils are involved in the recruitment and activation of T or B cells in RA. Neutrophils are an important source of TNF-α, which can induce production of CCL18 to recruit T cells to inflamed sites (64). Neutrophils can also release B-lymphocyte stimulator to activate B cells in RA (65, 66). In addition, delayed apoptosis of neutrophils, due to activation of anti-apoptotic signals and inhibition of pro-apoptotic pathways by various cytokines, contributes to the perpetuation of inflammation and development of RA (15). Moreover, neutrophils participate in the destruction of cartilage by stimulating synoviocytes to release MMPs and activating osteoclast through receptor activator of nuclear factor-kB ligand (RANKL) signaling (69–71). In this context, they can promote bone adsorption and inhibit bone remodeling in RA.

While neutrophils from peripheral blood of patients with active synovitis generate less ROS than those from normal donors and arthritis patients at clinical remission, those in the synovial fluid (SF) of patients display increased ROS production (97). This phenomenon may be explained by the recruitment of primed neutrophils with high ROS production from the blood to the joint during disease progression. These findings indicate that there are at least two subsets of neutrophils with different capability of ROS production in RA. Reduced production of cellular ROS in circulating neutrophils increases the susceptibility of autoimmune diseases and the risk of chronic inflammation, whereas elevation of ROS in SF neutrophils may directly participate in injury of joints tissues (67, 68). Although specific phenotype of these neutrophils in RA has not been defined, clearly there exist two different subsets of neutrophils with different ROS production, both of which are deleterious to patients possibly through distinct mechanisms in circulation and SF.

### Type 1 diabetes

Type 1 diabetes (T1D) is an autoimmune disease with a characteristic of immune-mediated destruction of pancreatic-β cells (98). T1D patients in prediabete stage or within 1 year after diagnosis show decreased number of neutrophils, and these cells increase to the normal level in those with T1D for more than 1 year (72). The reduction of neutrophils in T1D patients may be attributed, at least in part, to NETosis, that enhances formation of NETs and release of NE and proteinase 3 (PR3). The elevated levels of circulating NE and PR3 positively correlate to the increased seropositivity of autoantibodies reactive with β-cell antigen. NE and PR3 are also increased substantially even in autoantibody negative patients (72), suggesting that abnormal activity of neutrophil serine proteases may be involved in generation of autoantibodies and pathogenesis of disease at the early stage of T1D. However, a recent study reported that diabetic patients diagnosed within 3 years display decreased NE and PR3 levels (99). The discrepancies in the change of NE and PR3 may be caused by the differences in patient ages, disease severity, and stages. Therefore, the role of NETosis and its associated markers in the development of T1D need to be further investigated.

ROS, another important product of neutrophils, has also been reported to be elevated in patients with T1D (100),which may help initiate destruction of pancreatic β-cell (73). In addition, a number of cytokines produced by neutrophils, including IL-1, TNF-α, and IFN-γ, can also participate in the initiation of pancreatic-β cell destruction by stimulating production of toxic free radical or indirectly by inducing recruitment and activating of other immune cells (73). Since these cytokines are produced not only by neutrophils but also by other immune cell types and these immune cells are known to have pleiotropic and redundant effects, it is difficult to distinguish the role of individual cytokines in T1D. Other than their direct cytokine effect, the indirect effect of neutrophils on activation of other immune cells has also been documented. In non-obese diabetic (NOD) mice, DNA–anti-DNA IgG complexes stimulate formation of NETs and production of cathelicidin-related antimicrobial peptide (CRAMP). Subsequent activation of pDC and concurrent release of IFN-α promotes T cell-mediated autoimmune response at the early stage of T1D (16). Therefore, neutrophils in T1D appear to contribute to immune pathology by facilitating auto-antibody-mediated β-cell destruction and by enhancing inflammation. Given the heterogeneity of this cell population, it is intriguing to address the question as to whether anti-inflammatory and disease-protective neutrophils are present in T1D.

### Inflammatory bowel diseases

Inflammatory bowel diseases (IBD), including Crohn's disease (CD), and ulcerative colitis (UC), are characterized by chronic, relapsing inflammation in gastrointestinal tract, which are resulted from dysregulation of immune responses at intestinal mucosa (101). The role of neutrophils in the pathogenesis of IBD remains controversial. The neutrophil count in patients with IBD is significantly increased when compared to that in normal individuals (102). The number of infiltrating neutrophils is associated with the severity of UC (103). Patients with active UC often have a higher neutrophil-to-lymphocyte ratio than normal controls (104). These results suggest that neutrophils recruited to the lesion may be deleterious to the development of UC. Neutrophils can also be involved in regulation of disease development via MMP-9-mediated degradation of ECM. MMP-9 deficiency alleviates an inflammatory response and intestinal injury in DSS-induced IBD mice (76), and the inhibition of MMP-9 reduces disease severity (77). In addition, increased activity of NE in UC patients support potential involvement of NE in the pathogenesis of IBD (105). Over-reactive NE can disrupt intestinal barrier by degrading E-cadherins or zonula occludens-1 (79), and inhibit mucosal repair by suppressing proliferation of intestinal epithelial cells (80). However, defect of neutrophil recruitment contributes to the inefficient bacteria clearance and chronic inflammation in CD (106–108). Neutrophils from peripheral blood of CD patients produce increased ROS (109), which exhibit important antimicrobial properties known to protect host against microbial infections (74). However, over produced ROS can cause intestinal mucosal damage, thereby increasing mucosal permeability due to the degradation of polyunsaturated acids in the membrane of intestinal epithelial cells (75). Therefore, the suppression of ROS production, such as anti-TNF-α (infliximab) therapy, will be beneficial for IBD patients (110).

A recent study reported that CD177^+^ neutrophils exhibit a protective role against IBD. CD177 is a surface marker expressed exclusively on human neutrophils (111) and 45–60% of neutrophils in periphery blood of healthy individuals are CD177 positive (112). The CD177^+^ neutrophils have enhanced antimicrobial activity, which is associated with increased production of ROS, MPO, NETs as well as bactericidal peptides (17). Compared to CD177^−^ counterparts, CD177^+^ neutrophils display lower levels of pro-inflammatory cytokines, such as IFN-γ, IL-6, and IL-17A, but higher level of IL-22 (17), which promote restoration of epithelial integrity and resolution of colitis. CD177 knockout mice develop more severe colitis induced by DSS and compromised intestinal barrier integrity than wild-type mice (81). CD177 deficiency may impair accumulation of neutrophil to infected sites at the early stage of disease and therefore cause increased intestinal mucosa destruction (113). This association of neutrophils accumulation at the inflamed sites with CD177-dependent protection from colitis induced by DSS suggests these CD177^+^ neutrophils are anti-inflammatory and IBD-protective and appear to be distinct from pro-inflammatory LDGs found in SLE.

## Conclusion and future perspective

Neutrophils demonstrate multifaceted functions in cancers and autoimmune diseases, which are determined by their context dependent production of different effector molecules. These cells are generally divided into N1/N2 subsets in tumor site or alternatively HDNs/LDNs in peripheral blood of cancer patients. Although HDNs and LDNs can be distinguished based on their differences in density, currently there are no validated surface markers to phenotypically identify N1 and N2 neutrophils. However, LDGs in SLE and LDNs in cancer are functionally distinct, which raise a question of using density for neutrophils classification. It is clear that there are at least two subsets of neutrophils defined as immune-stimulatory/-suppressive or pro-/anti-inflammatory. These two subsets of neutrophils are highly plastic and can be skewed toward either direction, which is exemplified by type I interferon-driven polarization of immune-suppressive neutrophils into ones with an immunostimulatory phenotype.

Currently, neutrophils in autoimmune diseases do not have a clear classification. In most cases, they display a pro-inflammatory phenotype and promote the disease pathogenesis through multiple mechanisms (secretion of inflammatory molecules, activation of other immune cells, facilitate production of auto-antibodies). However, anti-inflammatory and disease-protective neutrophils have been identified in autoimmune conditions, including Gr-1^high^ neutrophils in EAE and CD177^+^ neutrophils in IBD, which supports the functional complexity of these heterogeneous immune cells. Although the tumor-promoting activity of N1 neutrophils has been well documented, little studies have been performed to target these cells for cancer therapy so far. A better understanding of these anti-inflammatory and disease-protective neutrophils associated with some autoimmune pathology may provide a potential new target for the treatment of these autoimmune disorders.

Understanding of the role of neutrophils is far from being complete. In the future, more research is needed to address the following questions. What are proper phenotypic markers to distinguish immuno-stimulatory/-suppressive or pro-/anti-inflammatory neutrophils? Are anti-inflammatory or immunosuppressive neutrophils exist broadly in all autoimmune diseases or are only present in specific autoimmune setting? Can TGF-β and type I interferons induce reciprocal polarization of pro- or anti-inflammatory neutrophils in the context of autoimmune diseases? What is the relationship between anti-inflammatory neutrophils in autoimmune diseases and immunosuppressive neutrophils in cancer? Can they be classified into the same subpopulation? Answers to these questions will provide important insights into the development and function of neutrophils in cancer and autoimmune diseases, which may lead to development of novel approaches to disease intervention.

## Author contributions

All authors listed have made a substantial, direct and intellectual contribution to the work, and approved it for publication. XW and LQ wrote the manuscript. HY, ZL, and X-YW revised the manuscript.

### Conflict of interest statement

The authors declare that the research was conducted in the absence of any commercial or financial relationships that could be construed as a potential conflict of interest.
